# *Ocimum tenuiflorum* extract (HOLIXER^TM^): Possible effects on hypothalamic–pituitary–adrenal (HPA) axis in modulating stress

**DOI:** 10.1371/journal.pone.0285012

**Published:** 2023-05-04

**Authors:** Mohan Gowda C. M., Sasi Kumar Murugan, Bharathi Bethapudi, Divya Purusothaman, Deepak Mundkinajeddu, Prashanth D’Souza

**Affiliations:** Department of Biology, R&D Centre, Natural Remedies Private Limited, Bengaluru, Karnataka, India; Nippon Medical School, JAPAN

## Abstract

*Ocimum tenuiflorum* is a sacred medicinal plant bestowed with multiple health benefits. This plant is traditionally considered an adaptogen. Many scientific studies have indicated the anti-stress potential of *Ocimum tenuiflorum* but with higher doses. The present study investigated the effects of Holixer^TM^ (a clinically studied standardized *Ocimum tenuiflorum* extract) on modulating stress using two *in vivo* models, namely the swim endurance study in mice and forced swim test in rats. In addition, we explored the mechanism of action of Holixer^TM^ on the HPA axis using two *in vitro* cell-based assays to check for its inhibitory effect on cortisol release and CRF1 receptor antagonistic activity. *Ocimum tenuiflorum* extract enhanced the swimming time in mice, reduced the stress-induced increase in immobility time, and prevented the increase in corticosterone in rats subjected to the forced swim test. Further, *Ocimum tenuiflorum* extract inhibited cortisol release and exhibited a significant CRF1 receptor antagonist activity. Thus, *Ocimum tenuiflorum* extract was found effective in managing stress, and the effect could be due to the inhibition of cortisol release and the antagonistic effect on the CRF1 receptors.

## 1 Introduction

Stress is an inevitable feeling that everyone undergoes in day-to-day life [1]. Stress is a normal physiological response that helps us to notice unusual or unexpected threats in and around us. Stressful situations either make people vulnerable or increase their capacity to deal with stressful situations depending on the duration of stress and the adaptogenic capacity of people. The term “stress” was first introduced by Hens Selye into the medical world to represent the effects of anything that seriously threatens homeostasis and is stated as “nonspecific response of the body to any demand [2, 3]. Acute stress (short-term extreme situations requiring a fight-or-flight response) is a survival strategy in healthy individuals [4–6]. However, prolonged unmanageable stress called chronic stress leads to many diseases like cardiovascular disease [7], diabetes [8, 9] and depression [8, 10]. Managing chronic stress involves the removal of stressors, gaining more knowledge about the stress, and taking supplements that help reduce the stress. Removing the stressor is not always possible (E.g., work stress). Gaining more knowledge about stress needs more time and effort, which could be a daunting task for many of us in today’s fast-paced world. Intake of supplements to reduce stress is a simple task and could get accomplished by the majority in today’s scenario. Indian tradition has left us with a medicinal plant that could reduce our stress in day-to-day life. Drinking water soaked with the leaves of this plant has been given as a “Prashad” in many Hindu temples to calm the people and make them peaceful during their short visits to these temples.

*Ocimum tenuiflorum* L. (Synonym: *Ocimum sanctum* L.) commonly known as Tulsi or Holy Basil is the plant used for managing stress. It is a sacred Indian medicinal plant with longstanding traditional use in multiple Indian systems of medicines such as Ayurveda, Siddha, and Unani [11]. *Ocimum tenuiflorum* has protected against iron-induced testicular toxicity by counteracting redox imbalance [12] and exhibited better seizure control, memory retention, oxidative stress reduction, and neuronal structure preservation [13]. It showed antistress effects in different animal models [14] and prevented a stress-induced decline in macrophage function [15]. In a human clinical study, the plant effectively managed symptoms of general stress when used at a dose of 1200 per day for six weeks [16]. Various phytochemicals, namely vicenin-2, orientin, cynaroside, betulinic acid, genistein with syringic acid, rosmarinic acid, eugenol, carnosic acid, oleanolic acid, ursolic acid, luteolin, and apigenin were confirmed to be present in *Ocimum tenuiflorum* [17]. Though available scientific literature indicated the antistress property of different extracts of *Ocimum tenuiflorum*, there are hardly any studies that evaluated the antistress property of *Ocimum tenuiflorum* extract at a low dose of ≤50 mg/kg body weight in mice. Therefore, the current study was aimed to evaluate the antistress property and the mechanism of action of a recently developed clinically studied [18] novel *Ocimum tenuiflorum* extract (Holixer^TM^) at lower doses.

## 2 Materials and methods

### 2.1 Plant material

*Ocimum tenuiflorum* plant material used in this study was obtained from a cultivated source and authenticated by the National Institute of Science Communication and Information Resources, New Delhi (Voucher number: RD-21872). The plant material was used to prepare a standardized extract of *Ocimum tenuiflorum* (developed and named Holixer^TM^ by M/s Natural Remedies Pvt. Ltd., Bangalore, India). The *Ocimum tenuiflorum* extract used in this study is a patent-pending (202241023540) hydroalcoholic extract derived from the leaves-rich aerial parts of *Ocimum tenuiflorum*. It was manufactured in a good manufacturing practice (GMP)-certified facility and is phytochemically standardized to ≥5%w/w (by High-Performance Liquid Chromatography) of the Ocimum Bioactive Complex. The extract complies with the United States Pharmacopeia (USP) requirements on microbial, heavy metals, residual pesticides, aflatoxins, and residual solvent limits for dietary supplements.

### 2.2 Chemicals and reagents

Human adreno-carcinoma (NCI-H295R) cell line and DMEM: F-12 Medium were obtained from ATCC. Corning^®^ ITS+ Universal Culture Supplement Premix and Corning Nu-Serum™ were used. Dimethyl sulfoxide (DMSO), forskolin, antalarmin hydrochloride, and ovine CRF were obtained from Sigma. Cortisol-HTRF Kit was obtained from Cisbio. Human recombinant CHO cell line was obtained from Invitrogen. Carboxy methyl cellulose (HiMedia Laboratories Pvt. Ltd., Mumbai, India) was used in this study.

### 2.3 Animals

Male Swiss albino mice of age 6–8 weeks were used in the swim endurance test, and male albino Wistar rats aged 6–8 weeks were used in the evaluation of the antistress activity of *Ocimum tenuiflorum* extract. All animals were maintained in a room at 22°C (±3°C), and relative humidity was kept between 30 and 70%. Artificial light was set to give a cycle of 12 hours light and 12 hours dark. UV-treated water and conventional laboratory rodent diet were offered ad libitum. Animals were adapted to the environment for at least one week before experiments. All the experimental procedures were approved by the Institutional Animal Ethics Committee (IAEC) of Natural Remedies Pvt Ltd, Bangalore, India (Approval no.: IAEC/NR-PCL-02/07/2021 & IAEC/NR-PCL-02/03/2020) and experiments were conducted according to the Committee for the Purpose of Control and Supervision of Experiments on Animals (CPCSEA) guidelines and in compliance with the Animal Research: Reporting of In Vivo Experiments (ARRIVE) guidelines.

### 2.4 Swim endurance test in mice

The swim endurance test was carried out using male *Swiss albino* mice aged 6–8 weeks [19, 20]. Animals were divided into four groups of 6 animals each and were housed with three animals per cage during the experiment. The body weight of each animal within a group was within ±20% of the group mean. Group 1 was administered orally with 0.5% w/v carboxy methyl cellulose (CMC) at a 10 ml/kg dose. *Ocimum tenuiflorum* extract was suspended at a specific concentration in 0.5% CMC and administered orally to groups 2, 3, and 4 at 12.5, 25, and 50 mg/kg body weight per day for ten days. On the 10^th^ day, one hour after administering the test substance, animals were subjected to the swim endurance test. Animals were made to swim in separate porcelain tanks of 30 cm in height and 70 cm in diameter filled with water maintained at 28–30°C. The endurance of each mouse was measured as swimming time recorded from the beginning of the time till exhaustion. A mouse was considered exhausted and rescued when it exhibited a loss of coordinated movements and failed to return to the water surface within a 10-s period to breathe for three consecutive times.

### 2.5 Antistress activity in the forced swim test

Animals were divided into five groups of 6 animals each and were housed with four animals per cage during the experiment. The body weight of each animal within a group was within ±20% of the group mean. Groups 1 and 2 were administered orally with 0.5% w/v carboxy methyl cellulose (CMC) at a dose of 10 ml/kg body weight for eight days. *Ocimum tenuiflorum* extract was suspended at a specific concentration in 0.5% CMC and administered orally to groups 3, 4, and 5 at a dose of 12.5, 25, and 50 mg/kg body weight respectively once a day for eight days. On the 8^th^ day, one hour after the administration of the test substance or vehicle, animals of groups 2 to 5 were subjected to the forced swim test [21]. Animals were allowed to swim for 5 minutes in a cylindrical tank constructed of transparent Plexiglas of 28 cm diameter and a height of 55 cm filled with water to a height of 40 cm. Animals were observed for immobility time as soon as they were subjected to the forced swim test. Immobility is characterized by the lack of movement except that which is necessary to keep the animal’s nose above the water level. Blood was collected from the retro-orbital plexus and centrifuged at 4000 g for serum separation, and corticosterone levels were determined in the serum using LC-MS/MS technique.

### 2.6 Cortisol release assay

#### 2.6.1 Cell culture and treatment

The cortisol release study was carried out to assess the effect of *Ocimum tenuiflorum* extract on cortisol secretion [22]. Human adreno-carcinoma (NCI-H295R) cells (No. CRL-2128) were grown in 75-cm^2^ flasks at 37°C with 5% CO_2_ in DMEM: F-12 Medium (No. 30–2006) containing 1% ITS+ Universal Culture Supplement Premix (No. 354352) and 2.5% Nu-serum supplement (No. 355100). The medium was changed three times a week. Before the start of the experiment, cells were subcultured from 80% confluent stock cultures into 48-well culture plates for 24 hours. After incubation, old media was replaced with fresh media (500 μl/well) containing the test sample (*Ocimum tenuiflorum* extract) at different non-cytotoxic concentrations from 6.25 μg/ml to 200 μg/ml and forskolin at 10 μM concentration. Dimethyl sulfoxide (DMSO) was used as the vehicle for the test sample and the concentration of DMSO in the test system was not more than 0.4%. After incubating for a period of 48 hours at 37°C in an atmosphere of 5% CO_2_, cell culture supernatant was removed from each well and frozen at -80°C for cortisol measurement.

#### 2.6.2 Cortisol assay

Cortisol concentration in the cell supernatant was determined using a cortisol-HTRF Kit [No. 64CRTPEB]. The assay was performed in triplicate. Briefly, 10 μL of the vehicle or sample was added to a 384 well plate. Cortisol-d2 (5 μL) and anti-cortisol-cryptate (5 μL) were added to all the wells except negative control for which 5 μL of detection buffer was used instead of the Cortisol-d2 reagent. The plate was sealed with a plate sealer and incubated for 2 h at room temperature in the dark. Reading was taken in a microplate reader (PHERAstar) at two wavelengths (665 and 620 nm). Percentage inhibition of cortisol by the test sample was calculated by considering the cortisol released by forskolin as 100%.

### 2.7 Antagonist effect in human CRF1 receptor

*Ocimum tenuiflorum* extract was tested for its antagonistic effect on CRF1 receptor [22, 23]. Before the treatment with test substances, human recombinant (CHO) cells (10,000 cells per well) were plated on 384-well, black-wall, clear-bottom assay plate containing growth medium and incubated for 20 h at 37°C and 5% CO_2_. After incubation, the cells were treated with *Ocimum tenuiflorum* extract at various non-cytotoxic concentrations ranging from 6.25 to 200 μg/ml for 30 minutes, followed by 30 minutes of incubation with ovine CRF (15 nM) at 37°C. DMSO was used as the solvent for the test substance, and cells treated only with the vehicle were used to get the control response. Antalarmin hydrochloride, a standard antagonist to the CRF1 receptor was used as the positive control. Levels of cAMP were determined in triplicate wells using HTRF detection method. Control (untreated but stimulated with CRF) cAMP level was considered a 100% response.

### 2.8 Statistical analysis

Data obtained in the studies were analyzed using one-way ANOVA followed by the Bonferroni method as the post–hoc test. In the case of heterogeneous data, the Dunnett T3 method was used after transformation. All values were reported as mean ± SD. Results were considered statistically significant if the p-value was less than 0.05.

## 3 Results

### 3.1 Swim endurance capacity of *Ocimum tenuiflorum* extract

**Fig 1:**
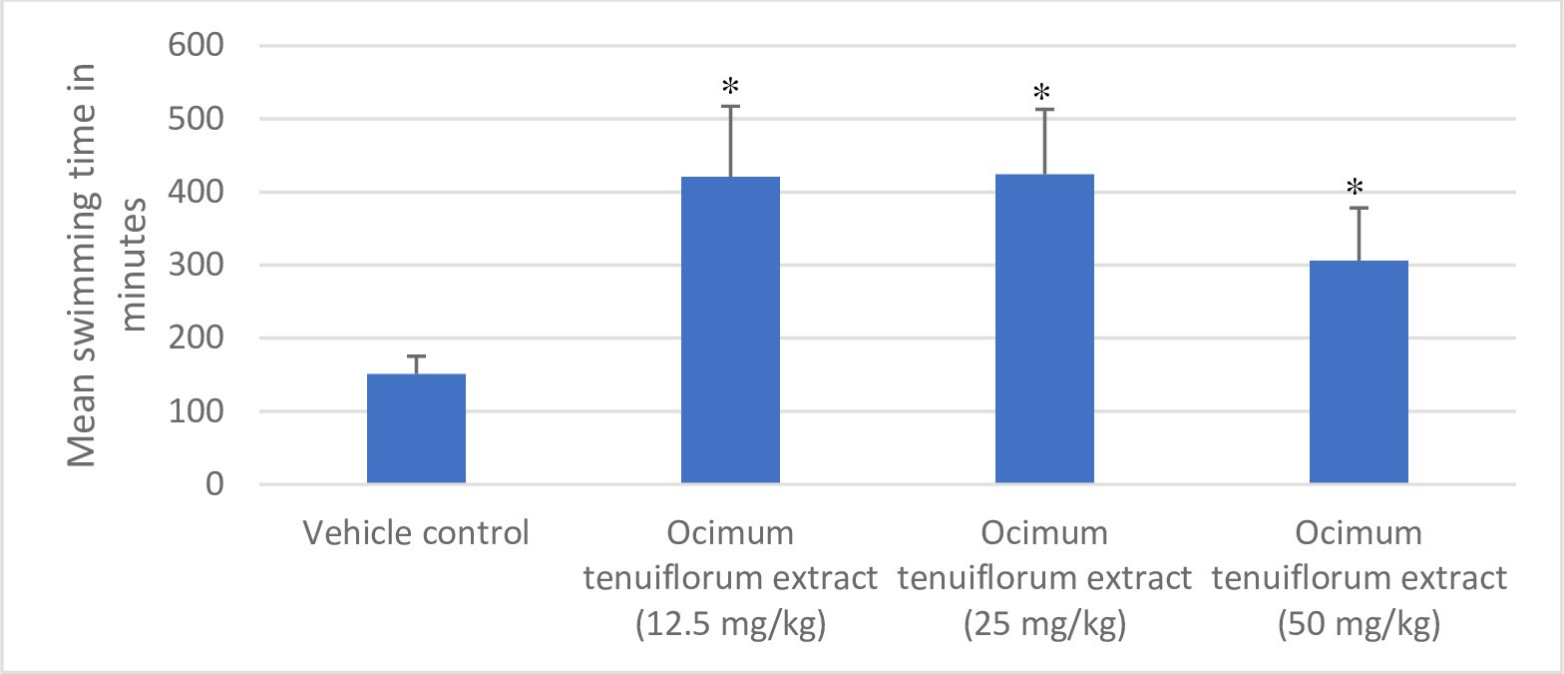
Effect of *Ocimum tenuiflorum* extract on swimming time in swim endurance study in mice. *Ocimum tenuiflorum* extract at 12.5, 25 and 50 mg/kg were evaluated using swim endurance test in mice. Swimming time till exhaustion was noted for each mouse and results presented as mean ± SD (n = 6). *p<0.05 compared to vehicle control. Higher the swimming time, better the endurance capacity of animals.

Results of the swim endurance test in mice are shown in Fig 1. *Ocimum tenuiflorum* extract at all the tested doses (12.5, 25, and 50 mg/kg) significantly enhanced the endurance capacity of mice compared to vehicle control mice measured in terms of the swimming time till exhaustion. *Ocimum tenuiflorum* extract at 12.5 mg/kg and 25 mg/kg bw showed maximum endurance enhancement in mice compared to the 50 mg/kg dose.

### 3.2 Antistress effect of *Ocimum tenuiflorum* extract

**Fig 2:**
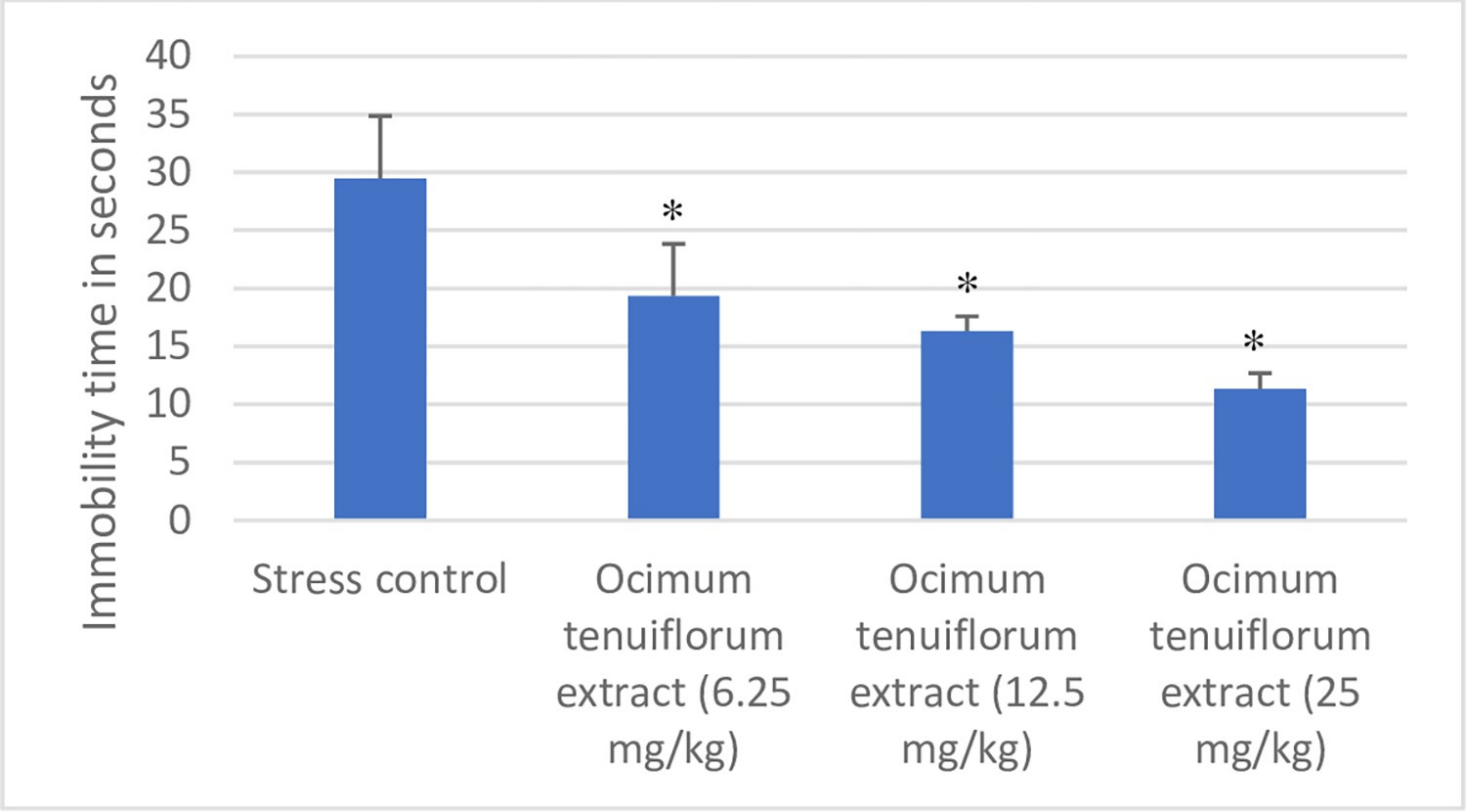
Effect of *Ocimum tenuiflorum* extract on immobility time of rats subjected to forced swim test. *Ocimum tenuiflorum* extract at 6.5, 12.5, and 25 mg/kg doses were evaluated in the forced swim test. The immobility time of animals during a 5-minute forced swim test was noted for each animal. Results are presented as mean ± SD (n = 6). *p<0.05 compared to stress control. Lesser the immobility time, the higher the antistress effect.

**Fig 3:**
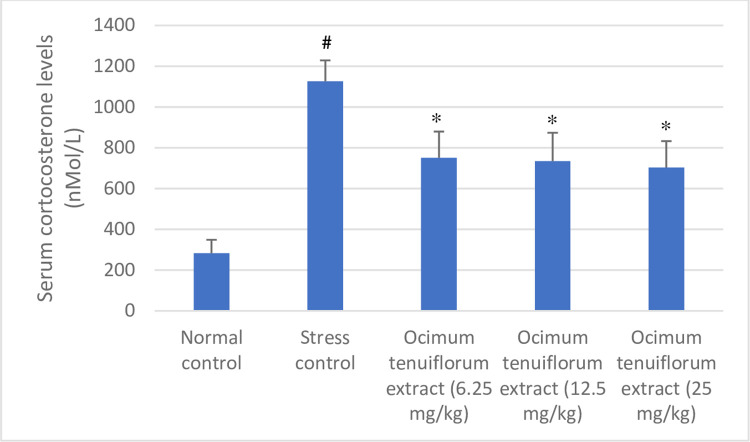
Effect of *Ocimum tenuiflorum* extract on serum corticosterone level of rats subjected to forced swim test. *Ocimum tenuiflorum* extract at 6.5, 12.5, and 25 mg/kg doses were evaluated in the forced swim test. Serum corticosterone levels were analyzed using the LCMS/MS technique. Results are presented as mean ± SD (n = 6). #p<0.05 compared to normal control. *p<0.05 compared to stress control. The lesser the serum corticosterone levels better the antistress effect.

The antistress effect of *Ocimum tenuiflorum* extract was studied using the forced swim test model. In addition to the immobility time, the serum corticosterone level was measured to understand the effect *of Ocimum tenuiflorum* extract on the stress hormone in rats. Results of *Ocimum tenuiflorum* extract in the forced swim test using rats are shown in Figs 2 and 3. Rats that got stressed on account of exposure to forced swimming tests and are unable to manage the forced swimming stress remain immobile for a longer time compared to rats that can manage the stress induced by forced swim test. *Ocimum tenuiflorum* extract showed a significant dose-dependent decrease in the immobility time from 6.25 mg/kg to 25 mg/kg dose compared to stress control rats indicating the ability of the extract to effectively manage the forced swim test stress. In addition, rats treated with *Ocimum tenuiflorum* extract successfully prevented the increase in serum level of corticosterone despite the stressful environment indicating its effectiveness.

### 3.3 Cortisol release assay

**Fig 4:**
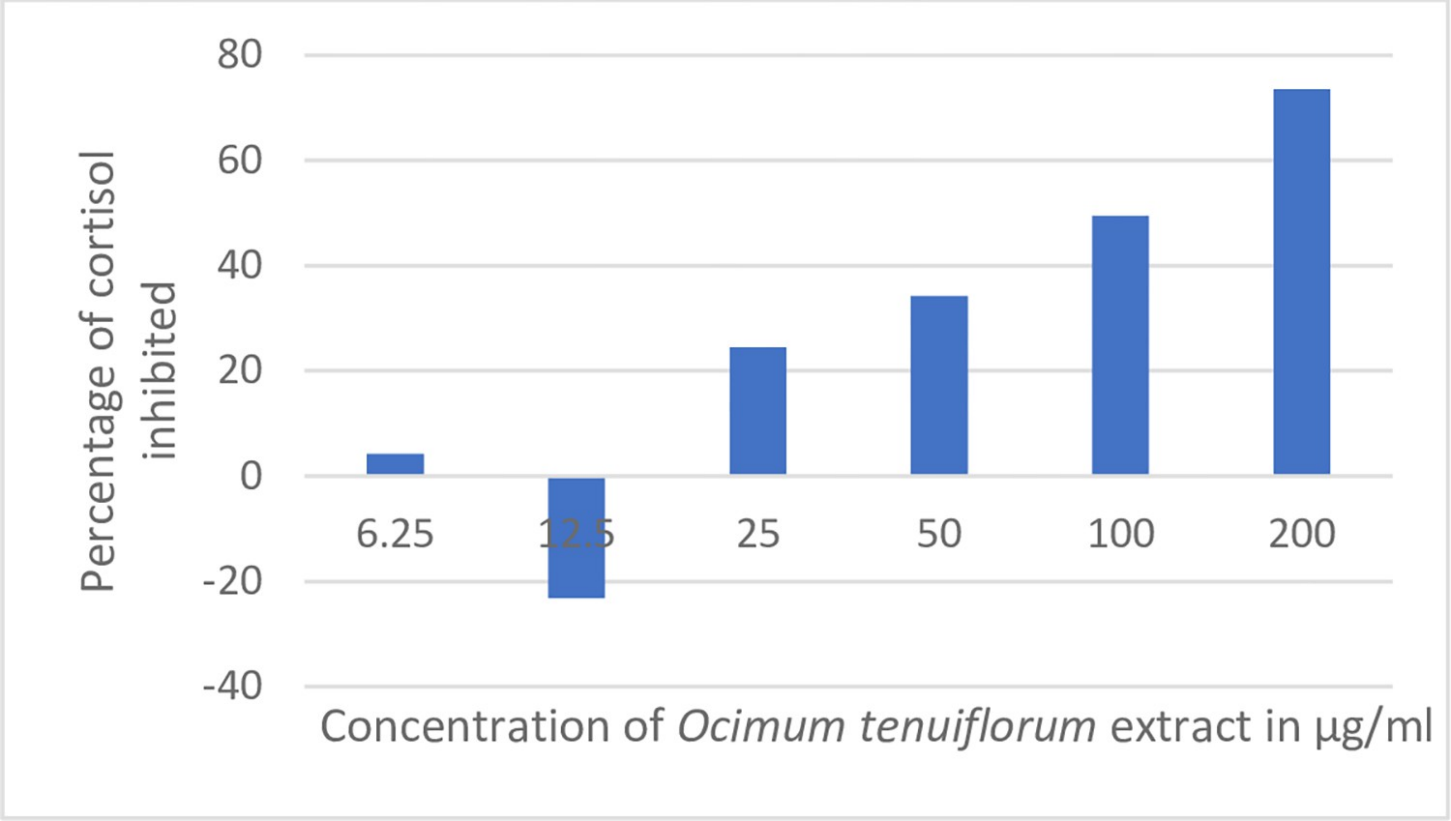
Effect of *Ocimum tenuiflorum* on the release of cortisol. *Ocimum tenuiflorum* extract at different non-cytotoxic concentrations was tested in the forskolin-induced cortisol release assay using human adreno-carcinoma (NCI-H295R) cells. The cortisol released by the cells was analyzed using a cortisol-HTRF kit, and the percentage of cortisol inhibited by the test substance is shown. The higher the percentage of cortisol inhibited, the better the activity.

*Ocimum tenuiflorum* was tested for its effect on forskolin-induced release of cortisol. Results of the effect of *Ocimum tenuiflorum* extract on cortisol are shown in Fig 4. *Ocimum tenuiflorum* showed a concentration-dependent reduction in the cortisol levels from 25 to 100 μg/ml. About 49.5% of cortisol was inhibited at 100 μg/ml concentration and 73.6% at 200 μg/ml concentration indicating the appreciable stress managing potential of *Ocimum tenuiflorum* extract.

### 3.4 CRF1 receptor assay

**Fig 5:**
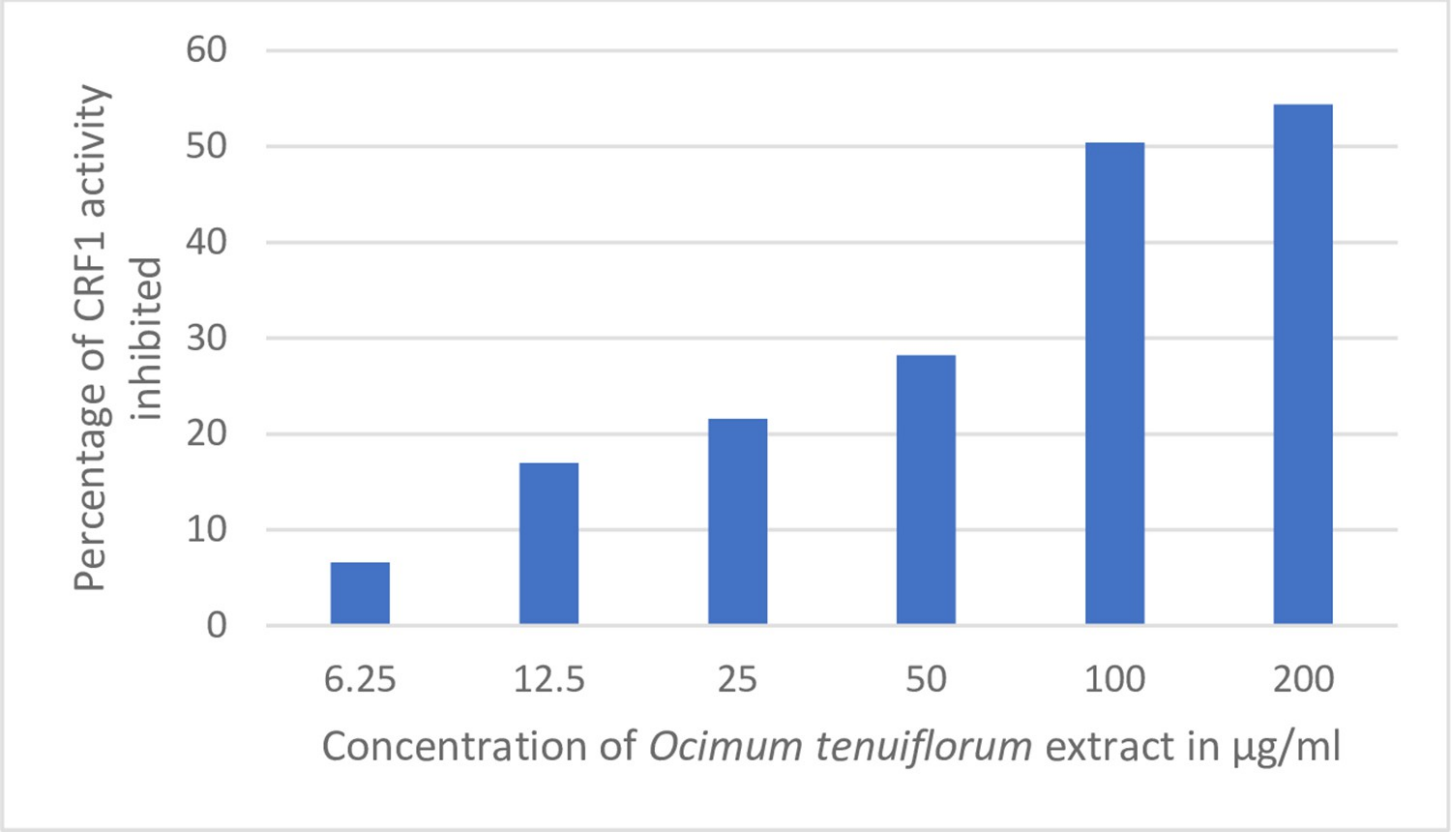
Antagonistic effect of *Ocimum tenuiflorum* extract with CRF1 receptor. *Ocimum tenuiflorum* extract at different non-cytotoxic concentrations was tested using human recombinant (CHO) cells containing CRF1 receptors. The antagonistic effect was assessed by analyzing the levels of cAMP using a HTRF detection method. Reduction in the levels of cAMP is presented as the percentage of CRF1 activity inhibited. The higher the percentage of CRF1 activity inhibited, the better the antagonistic effect.

The CRF1 receptor assay was conducted to see if the cortisol inhibition property of *Ocimum tenuiflorum* could be mediated through antagonism at the CRF1 receptor. Results of the CRF1 activity are shown in Fig 5. *Ocimum tenuiflorum* extract inhibited the CRF1 activity by 50.4% at 100 μg/ml. This antagonistic effect of *Ocimum tenuiflorum* extract against the CRF1 receptor could partly be responsible for the cortisol inhibition property of *Ocimum tenuiflorum* extract.

## 4 Discussion

Multiple scientific studies indicate the usefulness of cortisol as a biomarker of stress in humans [24–26]. Chronic stress affects the functions of the nervous system; immune system; cardiovascular system; gastrointestinal system; and endocrine system [27]. Cortisol is a stress hormone secreted by the human body in response to stress; homeostatic regulatory mechanisms maintain cortisol levels within a narrow range; the hypothalamus-pituitary-adrenal axis (HPA) regulates the release of cortisol. During stressful situations, the HPA axis gets activated and releases the corticotrophin-releasing hormone (CRH) from the hypothalamus. The CRH helps in the release of adrenocorticotrophic hormone (ACTH) by binding with the corticotrophin-releasing hormone receptor 1 (CRF1) of the anterior pituitary gland, and the ACTH stimulates the adrenal glands to secrete cortisol helping the body to combat stress [28, 29]. When cortisol level in the blood reaches a particular threshold, it inhibits the release of CRH from the hypothalamus and is called the negative feedback mechanism. Cortisol provides more energy to the body by increasing the blood glucose level and is used to combat stress. Cortisol increases blood glucose levels by breaking down the protein and fat stores. Thus, the HPA axis protects the body during stressful situations and is a normal physiological function. However, long-term repeated activation of the HPA-axis results in the dysfunction of the negative feedback mechanism leading to increased cortisol levels in the blood, and a higher level of blood cortisol for a prolonged duration in people with chronic stress may lead to obesity [30], poor immunity [31], diabetes [30], cardiovascular disease [32–34], osteoporosis [35], poor memory [36], and depression [36]. Thus, cortisol is considered a primary target of substances that reduce chronic stress, and reducing cortisol levels to a near-normal level is the goal of reducing the risk of subsequent health implications.

*Ocimum tenuiflorum* is an adaptogenic herb with multiple health benefits. Therefore, we evaluated its stress managing properties using swim endurance test in mice, and forced swim test in rats, cortisol release assay *in vitro* and CRF1 antagonistic effect *in vitro*. Several studies have already shown the antistress property of *Ocimum tenuiflorum*. Ethanol extract from the leaves of *Ocimum tenuiflorum* showed antistress effects in both acute stress and chronic unpredictable stress models at a dose of 200 mg/kg body weight in rats. The extract was reported to have significantly prevented the stress induced increase in plasma corticosterone levels [37].

The phytochemical constituents that are analysed as part of Ocimum Bioactive Complex (patent-pending), including rosmarinic acid and luteolin, was hypothesized to contribute to the antistress effect of Holixer^TM^. The individual phytoconstituents of OBC have been well researched for its various pharmacological activity, in particular the anti-stress effect. Rosmarinic acid has been reported to reduce plasma corticosterone levels and increase the expression of hippocampal mineralocorticoid receptors, glucocorticoid receptors and brain-derived neurotrophic factor in the hippocampus of rats induced with HIV-1 viral protein tat injection and subsequent repetitive restrain stress [38]. Rosmarinic acid has also been reported to reduce maternal stress induced corticosterone increase [39], chronic unpredictable stress induced increase in corticosterone [40] and reverse stress induced changes of SUV39H1, Mkp-1, and BDNF expression in the mice hippocampus [41]. Luteolin-7-O-glucuronide, another constituent of OBC, has been found to reduce blood corticosterone in a mouse sleep deprivation model, wherein the compound improved depression-like and stress coping behaviours [42].

An ethanol extract of leaves of *Ocimum sanctum* was reported to prevent the noise-stress induced increase in corticosterone in Wistar rats at an intraperitoneal dose of 100 mg/kg body weight [43]. When restraint-stressed rats, were evaluated in elevated plus maze test, tail suspension test, and forced swimming test after treatment with ethanol extract of *Ocimum tenuiflorum* leaves, 200 mg/kg body weight dose had better antistress effects [44]. Aqueous extract of *Ocimum tenuiflorum* leaves at a dose of 100 mg/kg body weight has shown antistress effects in rats subjected to restraint stress [14, 45]. Aqueous leaf extract of *Ocimum tenuiflorum* was found to reduce stress-induced gastric ulcers in rats at a dose of 100 and 200 mg/kg body weight [46]. The antistress activity was also reported for the methanol extract of the whole plant of *Ocimum tenuiflorum* extract at doses from 50 to 200 mg/kg body weight in rats subjected to chronic variable stress [22]. The same extract was found to show CRF1 antagonist activity of 63% at 100 μg/ml concentration and cortisol release inhibition of 89% at 100 μg/ml concentration. Methanol extract of the roots of *Ocimum sanctum* was reported to increase swimming time in mice at an intraperitoneal dose of 400 mg/kg body weight [47]. Though there are many studies available on the antistress activity of *Ocimum tenuiflorum*, no animal studies are available on the extract at lower doses from 6.25 mg/kg to 50 mg/kg body weight in rats or mice.

In line with previously reported studies of *Ocimum tenuiflorum*, this study confirmed the antistress potential of *Ocimum tenuiflorum* but at lower doses in mice and rats, which substantiates the improved efficacy of the extract used in this study. The antistress activity was also substantiated by its stress hormone lowering effect observed in the cortisol release assay and antagonism at the CRF1 receptor. The results presented in this study serve as a plausible mechanism of the stress management property of the *Ocimum tenuiflorum* extract (Holixer^TM^) observed in the recent 8-week randomized double-blind placebo-controlled study [18]. In the clinical study, the *Ocimum tenuiflorum* extract (Holixer^TM^) significantly reduced stress as measured by the Perceived Stress Scale (PSS), improved sleep as measured by the Athens Insomnia Scale (AIS), and reduced hair cortisol in adults experiencing stress at a daily dose of 250 mg/day.

This study also has few limitations. We could have subjected the animals to chronic stress paradigm before subjecting them to forced swim test that might have produced additional data in chronic stressful condition. Also, adaptogenic biomarkers such as molecular chaperons (e.g., Hsp70), stress-activated c-Jun N-terminal protein kinase (JNK1), Forkhead box O (FoxO) transcription factor, and nitric oxide (NO) would have been a value addition.

## 5 Conclusion

Overall, the results demonstrated that the standardized extract of *Ocimum tenuiflorum* exhibits antistress benefits at a very low dose as evident in the two *in vivo* studies and its antistress activity could be due to the inhibition of cortisol release and antagonistic effect on the CRF1 receptors thereby regulating the HPA axis. The antistress effect of Holixer^TM^ at lower dose has been confirmed in the recent clinical study in subjects experiencing stress.

## Supporting information

S1 Table Minimal data set for figures. (DOCX)
