# Correction: *Ocimum tenuiflorum* extract (HOLIXER^TM^): Possible effects on hypothalamic–pituitary–adrenal (HPA) axis in modulating stress

**DOI:** 10.1371/journal.pone.0344461

**Published:** 2026-03-05

**Authors:** Mohan Gowda C. M., Sasi Kumar Murugan, Bharathi Bethapudi, Divya Purusothaman, Deepak Mundkinajeddu, Prashanth D’Souza

In the 2.5 Antistress activity in the forced swim test subsection of the 2 Materials and methods, there is an error in the fourth sentence of the paragraph. The correct sentence is: *Ocimum tenuiflorum* extract was suspended at a specific concentration in 0.5% CMC and administered orally to groups 3, 4, and 5 at a dose of 6.25,12.5, and 25 mg/kg body weight respectively once a day for eight days.

In the Discussion, there is an error in the first sentence of the third paragraph. The correct sentence is: The phytochemical constituents that are analysed as part of Ocimum Bioactive Complex (patent-pending), including rosmarinic acid and luteolin-7-O-glucuronide, was hypothesized to contribute to the antistress effect of Holixer^TM^.
