# Supplementary material for: Ocimum tenuiflorum extract (HOLIXERTM): Possible effects on hypothalamic–pituitary–adrenal (HPA) axis in modulating stress
Source: PLoS One. 2023 May 4;18(5):e0285012. doi: 10.1371/journal.pone.0285012 (PMC10159140; doi:10.1371/journal.pone.0285012)
Supplement: S1 Table — (DOCX) [file pone.0285012.s001.docx]

**S1 Table: Minimal data set for figures**

| **Fig. #** | **Mean** | **S.D** | **S.E** | **Statistical method used** | **P value** | **# samples** |
| --- | --- | --- | --- | --- | --- | --- |
| **Fig 1** |  |  |  | One-way repeated measures ANOVA/Bonferroni: compare selected pairs | *P <0.05 |  |
| Vehicle Control | 151.33 | 23.96 | 9.78 |  |  | 6 |
| Ocimum tenuiflorum extract (12.5 mg/kg) | 420.83 | 96.53 | 39.41 |  |  | 6 |
| Ocimum tenuiflorum extract (25 mg/kg) | 424.33 | 88.67 | 36.20 |  |  | 6 |
| Ocimum tenuiflorum extract (50 mg/kg) | 305.83 | 72.28 | 29.51 |  |  | 6 |
| **Fig 2** |  |  |  | One-way repeated measures ANOVA/Bonferroni: compare selected pairs | *P <0.05 |  |
| Stress Control | 29.5 | 5.36 | 2.19 |  |  | 6 |
| Ocimum tenuiflorum extract (6.25 mg/kg) | 19.33 | 4.50 | 1.84 |  |  | 6 |
| Ocimum tenuiflorum extract (12.5 mg/kg) | 16.33 | 2.16 | 0.88 |  |  | 6 |
| Ocimum tenuiflorum extract (25 mg/kg) | 11.33 | 1.36 | 0.56 |  |  | 6 |
| **Fig 3** |  |  |  | One-way repeated measures ANOVA/Bonferroni: compare selected pairs | *P <0.05 |  |
| Normal control | 282.64 | 66.06 | 26.96 |  |  | 6 |
| Stress Control | 1126.27 | 102.28 | 41.75 |  |  | 6 |
| Ocimum tenuiflorum extract (6.25 mg/kg) | 730.90 | 128.35 | 52.59 |  |  | 6 |
| Ocimum tenuiflorum extract (12.5 mg/kg) | 734.61 | 139.07 | 56.76 |  |  | 6 |
| Ocimum tenuiflorum extract (25 mg/kg) | 703.5 | 129.1 | 52.69 |  |  | 6 |
| **Fig 4** |  |  |  |  |  |  |
| Ocimum tenuiflorum extract (6.25 µg/ml) | 4.21 | 0.227 | 0.131 |  |  | 3 |
| Ocimum tenuiflorum extract (12.5 µg/ml) | -23.18 | 9.935 | 5.736 |  |  | 3 |
| Ocimum tenuiflorum extract (25 µg/ml) | 24.47 | 9.215 | 5.320 |  |  | 3 |
| Ocimum tenuiflorum extract (50 mg/kg) | 34.30 | 7.361 | 4.250 |  |  | 3 |
| Ocimum tenuiflorum extract (100 µg/ml) | 49.45 | 7.500 | 4.330 |  |  | 3 |
| Ocimum tenuiflorum extract (200 µg/ml) | 73.60 | 0.495 | 0.286 |  |  | 3 |
| **Fig. 5** |  |  |  |  |  |  |
| Ocimum tenuiflorum extract (6.25 µg/ml) | 6.6 | 6.248 | 3.607 |  |  | 3 |
| Ocimum tenuiflorum extract (12.5 µg/ml) | 17 | 10.312 | 5.953 |  |  | 3 |
| Ocimum tenuiflorum extract (25 µg/ml) | 21.6 | 2.902 | 1.676 |  |  | 3 |
| Ocimum tenuiflorum extract (50 mg/kg) | 28.2 | 2.300 | 1.328 |  |  | 3 |
| Ocimum tenuiflorum extract (100 µg/ml) | 50.4 | 4.692 | 2.709 |  |  | 3 |
| Ocimum tenuiflorum extract (200 µg/ml) | 54.4 | 8.005 | 4.622 |  |  | 3 |
